# Depression and Objectively Measured Physical Activity: A Systematic Review and Meta-Analysis

**DOI:** 10.3390/ijerph17103738

**Published:** 2020-05-25

**Authors:** Vincenza Gianfredi, Lorenzo Blandi, Stefano Cacitti, Mirko Minelli, Carlo Signorelli, Andrea Amerio, Anna Odone

**Affiliations:** 1 School of Medicine, Vita-Salute San Raffaele University, 20132 Milan, Italy; signorelli.carlo@hsr.it (C.S.); odone.anna@hsr.it (A.O.); 2 Accademia Lombarda di Sanità Pubblica, Consorzio Pavese Studi Post-Universitari, Unit of Forensic Medicine and Forensic Sciences, Department of Public Health, Experimental and Forensic Medicine, University of Pavia, 27100 Pavia, Italy; lorenzo.blandi01@universitadipavia.it (L.B.); stefano.cacitti01@universitadipavia.it (S.C.); mirko.minelli01@universitadipavia.it (M.M.); 3 CAPHRI Care and Public Health Research Institute, Maastricht University, 6211 Maastricht, The Netherlands; 4 Unit of Forensic Medicine and Forensic Sciences, Department of Public Health, Experimental and Forensic Medicine, University of Pavia, 27100 Pavia, Italy; 5 Department of Neuroscience, Rehabilitation, Ophthalmology, Genetics, Maternal and Child Health (DINOGMI), Section of Psychiatry, University of Genoa, 16132 Genoa, Italy; andrea.amerio@unige.it; 6 IRCCS Ospedale Policlinico San Martino, 16132 Genoa, Italy; 7 Mood Disorders Program, Tufts Medical Center, Boston, MA 02111, USA

**Keywords:** depressive symptoms, depression, physical activity, accelerometer, objectively measure, meta-analysis

## Abstract

Depression is a major contributor to the overall global burden of disease, with high prevalence and relapse rate. Several factors have been considered in order to reduce the depression burden. Among them, physical activity (PA) showed a potential protective role. However, evidence is contrasting probably because of the differences in PA measurement. The aim of this systematic review with meta-analysis is to assess the association between objectively measured PA and incident and prevalent depression. The systematic review was conducted according to methods recommended by the Cochrane Collaboration and the Preferred Reporting Items for Systematic Reviews and Meta-Analyses (PRISMA) guidelines. Relevant papers published through 31 August 2019 were identified searching through the electronic databases PubMed/MEDLINE, Excerpta Medica dataBASE (Embase), PsycINFO, Scopus, Web of Science (WoS), and the Cochrane Library. All analyses were conducted using ProMeta3. Finally, 42 studies met inclusion criteria. The overall Effect size (ES) of depression for the highest vs. the lowest level of PA was −1.16 [(95% CI = −1.41; −0.91), *p*-value < 0.001] based on 37,408 participants. The results of the meta-analysis showed a potential protective effect of PA on prevalent and incident depression.

## 1. Introduction

Depression is one of the major leading causes of disability worldwide, affecting approximately 400 million people [1], with 9% of men and 17% of women experiencing depressive symptoms at least once in their life. Mainly due to social prejudices, depression continues to be frequently under-diagnosed and inadequately treated [2]. Depression can have several negative consequences, being characterized by sad mood and/or loss of interest, affecting thoughts, feelings, behaviors, physical health and impairing social and occupational functioning [3,4]. Furthermore, over 80% of depressed patients have more than one depressive episode during their lifespan [5,6]. In this context, innovative and effective preventive and therapeutic strategies are required.

Current studies are focusing on the important role played by lifestyles and in particular physical activity (PA), in both preventing and treating depression [7]. Several biological mechanisms are potentially involved in the association between PA and depression, such as the noradrenergic and serotoninergic effects of physical activity [8], the hypothalamic-pituitary-adrenal axis regulation [9], the production of neurotrophic factor [10], and lastly the improvement in vascular function and oxygenation [11,12]. However, despite the high number of potential explanations, evidence is not concordant in proven positive association between PA and depression, for both prevention and treatment. One of the main reasons behind these contrasting results could be the different methods used to measure physical activity.

Two recent meta-analyses focusing on prevalent depression and incident depression found an inverse association between prevalent depression and PA [13], while this association was not significant when incident depression has been considered [14]. However, the study conducted by Schuch et al. retrieved only one paper using the objectively measured PA [13]. The meta-analysis conducted by Krogh et al. included trials that prescribed different types of exercise sessions without objectively measuring PA [14]. On the other hand, growing evidence is focusing on objectively measured physical activity, using for instance accelerometer and pedometer, showing how objectively measured PA is more precise than self-reported one. This was particularly true in estimating duration, total amount and intensity [15].

We performed a systematic review with meta-analysis of the evidence from the literature to assess the relation between physical activity objectively measured and incident and prevalent depression.

## 2. Materials and Methods

We conducted this systematic review according to the methods recommended by the Cochrane Collaboration [16] and to the Meta-analysis Of Observational Studies in Epidemiology (MOOSE) guidelines [17] and documented the process and results in accordance with the Preferred Reporting Items for Systematic Reviews and Meta-Analyses (PRISMA) guidelines [18]. The review protocol has been registered on PROSPERO [19], the International Prospective Register of Systematic Reviews funded by the National Institute of Health Research (https://www.crd.york.ac.uk/prospero/).

### 2.1. Information Sources and Search Strategy

Studies were identified searching through the electronic databases PubMed/MEDLINE, Embase, Scopus, Web of Science (WoS), PsycINFO and the Cochrane Library. We combined the search strategy of free text terms and exploded MESH headings for the topics of depression, physical activity, objective measurements, and type of study. The strategy was firstly developed in PubMed/MEDLINE and then adapted for use in the other databases (Supplementary Table S1). Studies conducted on human subjects and published in English through 31 August 2019 were included.

### 2.2. Inclusion and Exclusion Criteria

We considered studies that investigated the relation between physical activity objectively measured and depression, both as a continuous and as a binary variable. Adult participants of both sexes were considered. As done before [20,21], both population-based and hospital-based studies were included. Among hospital-based studies, inpatients, day-hospital, and outpatient subjects were included while emergency care records were excluded as considered non-representative. All experimental and observational study designs were included apart from case reports. Narrative and systematic reviews, letters to the editor and book chapters were excluded. Table 1 shows a detailed description of inclusion/exclusion criteria according to the Population, Exposure, Outcomes and Study design (PEOS) [22], adjusted for observational studies extended with time and language filters, as recommended by the Cochrane Collaboration [16].

### 2.3. Study Selection and Data Extraction

Identified studies were independently reviewed for eligibility by two couples of authors (VG, LB, MM, SC) in a two-step process: a first screening was performed based on title and abstract, while full texts were retrieved for the second screening. At both stages disagreements by reviewers were resolved by consensus. Data were independently extracted by three authors (LB, MM, SC) and supervised by a senior author (VG) using an ad-hoc developed data extraction spreadsheet. The data extraction spreadsheet was piloted on 10 randomly selected papers and modified accordingly. As done before [23,24,25], both qualitative and quantitative data was extracted from the original studies. Qualitative data recorded included the following items: name of first author and year of publication, country where the study was conducted and period during which the study was performed, device used to measure PA and tool used for depression diagnosis. Moreover, characteristics of the subjects were recorded (e.g., age, gender, comorbidities). Quantitative data extracted includes: sample size, number of participants lost (attrition), duration of PA measurement, distribution of depressed participants in the sample, level of PA performed and the results estimating the association between PA objectively measured and depression.

### 2.4. Quality Evaluation

The quality evaluation of the included publications were independently assessed by two authors using the New-Ottawa Scale [26] for observational studies and Cochrane Collaboration tool for trials [27].

### 2.5. Meta-Analysis

We pooled individual studies data using ProMeta3^®^ (Internovi, Milano, Italy) software. Due to heterogeneity, a random effects meta-analysis was employed. In order to reduce the heterogeneity, two sensitivity analyses were conducted, considering the following items: (i) study design, (ii) participants’ comorbidities. Moreover, a subgroup analysis by gender was conducted in order to estimate potential different effects among the two groups. We assessed publication bias with the visual inspection of a funnel plot [27] and the Begg [28] and Egger [29] tests.

## 3. Results

### 3.1. Literature Search

**Figure 1:**
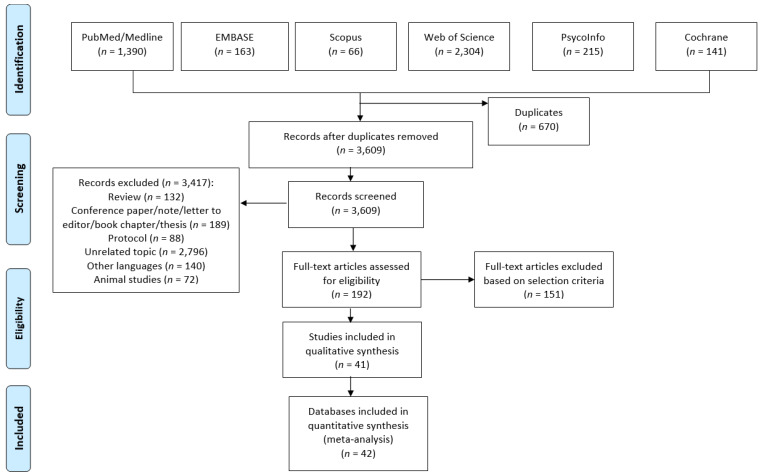
Flow diagram of the selection process.

A total of 4279 articles were retrieved. After a preliminary screening 670 articles were excluded because of duplicates, 409 not original papers (reviews, letters to the editor, editorials, protocols, etc.), and 2796 covering a different topic. After title and abstract screening, a total of 192 full-text articles were consulted, while at the end of the screening process only 41 were included in the systematic review [30,31,32,33,34,35,36,37,38,39,40,41,42,43,44,45,46,47,48,49,50,51,52,53,54,55,56,57,58,59,60,61,62,63,64,65,66,67,68,69,70]. As it was not possible to extrapolate data from one study, it was not included in the quantitative evaluation [67]. Figure 1 shows the selection process. Two studies reported separate data for men and women [49,54] and for this reason they were considered separately, resulting in 42 datasets being included in the meta-analysis.

The characteristics of the included studies are reported in Table 2. The majority of the studies were conducted in Europe (*n* = 18, 43%) and North America (*n* = 12, 29%). The first study assessing objectively measure PA and depression was published in 2004 [68]. The smallest sample size included in a study was of 23 participants [70], whereas the largest sample size was of 16,415 participants [62]. Twenty-six of the 42 datasets were cross-sectional (62%), eight trials (19%), six cohort studies (14%), and one case-control study (2%). The quality assessment of trials is reported in Supplementary Table S2. Thirty-two datasets (76%) used an accelerometer as the measurement device, while nine datasets (21%) used a pedometer. In almost all studies participants were asked to wear the device for 7 days, and even in cohort studies PA was measured only at baseline. With regard to depression, heterogeneous tools were used to make diagnosis, such as the Hospital Anxiety and Depression Scales (HADS), the Patient Health Questionnaire-9 (PHQ-9), the Beck Depression Inventory (BDI-II) and the Center for Epidemiologic Studies Depression Scale (CESD). Most of the time HADS was used (*n* = 11), followed by PHQ-9 questionnaire (*n* = 9); however almost all studies used a validated tool. At the same time, the results were expressed using different measures, as for instance Odd Ratio (OR), Relative Risk (RR), β coefficient (β) and Spearman’s Rho (r). 

### 3.2. Results of Meta-Analysis

**Figure 2:**
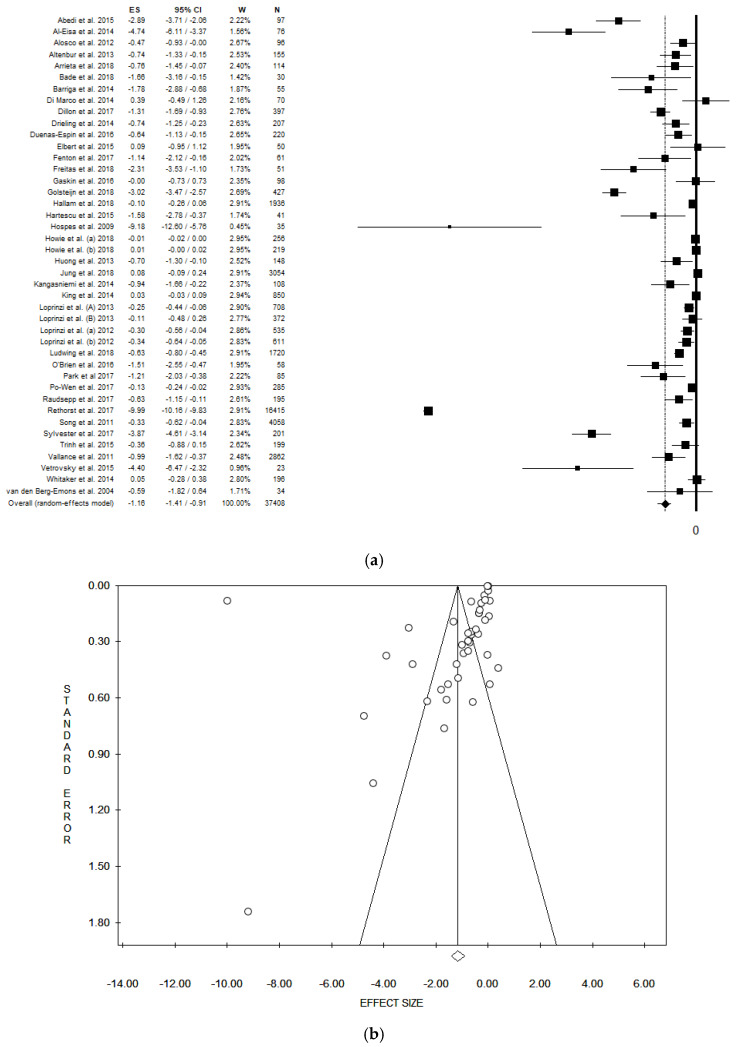
(**a**) Forest plot, (**b**) funnel plot and of the meta-analysis assessing the association between physical activity and depression. ES, effect size; CI, confidence interval.

The pooled ES was −1.16 [(95% CI = −1.41; −0.91), *p*-value < 0.001] based on 37,408 participants (Figure 2a), with high statistical heterogeneity (Chi^2^ = 15,090.18, df = 41, I^2^ = 99.73, *p*-value < 0.001). A potential publication bias was found by the visual assessment of the funnel plot and confirmed by the Egger’s linear regression test (Intercept −5.85, t = −1.91, *p*-value = 0.063). However, the ES estimated did not change after the trim and fill method (Figure 2b).

### 3.3. Sensitivity Analysis by Participants’ Comorbidities

The sub-group analysis considering only the general population (without diseases), included 21 datasets, and the pooled ES was −1.32 [(95% CI = −1.67; −0.97), *p*-value < 0.001] based on 33,812 subjects. High statistical heterogeneity was found (Chi^2^ = 14,715.47, df = 20, I^2^ = 99.86, *p*-value < 0.001). However, no publication bias was found by the visual assessment of the funnel plot and confirmed by the Egger’s linear regression test (Intercept −9.46, t = −1.50, *p*-value = 0.150). The sub-group analysis considering patients with chronic obstructive pulmonary disease (COPD), included 6 datasets, and the pooled ES was −1.08 [(95% CI = −1.91; −0.24), *p*-value = 0.012] based on 683 subjects. High statistical heterogeneity was found (Chi^2^ = 33.35, df = 5, I^2^ = 85.01, *p*-value < 0.001). However, no publication bias was found by the visual assessment of the funnel plot and confirmed by the Egger’s linear regression test (Intercept −4.12, t = −2.06, *p*-value = 0.109). The sub-group analysis considering obese participants, included 5 datasets, and the pooled ES was −0.35 [(95% CI = −0.80; 0.10), *p*-value = 0.128] based on 1354 participants. High statistical heterogeneity was found (Chi2 = 22.86, df = 4, I^2^ = 82.50, *p*-value < 0.001). However, no publication bias was found by the visual assessment of the funnel plot and confirmed by the Egger’s linear regression test (Intercept −1.86, t = −1.65, *p*-value = 0.197). The sub-group analysis considering participants with (any type of) cancer, included 5 datasets, and the pooled ES was −1.79 [(95% CI = −3.35; −0.22), *p*-value = 0.025] based on 955 participants. High statistical heterogeneity was found (Chi^2^= 112.21, df = 4, I^2^ = 96.44, *p*-value < 0.001). However, no publication bias was found by the visual assessment of the funnel plot and confirmed by the Egger’s linear regression test (Intercept 1.27, t = 0.16, *p*-value = 0.885).

### 3.4. Sensitivity Analysis by Study Design

The sub-group analysis considering only observational studies (cross-sectional, cohort and case-control studies), included 34 datasets, and the pooled ES was −0.99 [(95% CI = −1.26; −0.72), *p*-value < 0.001] based on 34,764 participants. High statistical heterogeneity was found (Chi^2^ = 14,809.58, df = 33, I^2^ = 99.78, *p*-value < 0.001). However, no publication bias was found by the visual assessment of the funnel plot and confirmed by the Egger’s linear regression test (Intercept −6.13, t = −1.61, *p*-value = 0.118). The sub-group analysis considering only cross-sectional analysis, included 27 datasets, and the pooled ES was −0.23 [(95% CI = −0.30; −0.16), *p*-value < 0.001] based on 17,191 participants. A high statistical heterogeneity was found (Chi^2^ = 240.33, df = 26, I^2^ = 89.18, *p*-value < 0.001). A publication bias was found by the visual assessment of the funnel plot and confirmed by the Egger’s linear regression test (Intercept −2.25, t = −4.89, *p*-value < 0.001). The sub-group analysis considering only cohort datasets, included 6 datasets, and the pooled ES was −2.61 [(95% CI = −7.41; 2.21), *p*-value < 0.289] based on 17,515 participants. High statistical heterogeneity was found (Chi^2^ = 10105.57, df = 5, I^2^ = 99.95, *p*-value < 0.001). However, no publication bias was found by the visual assessment of the funnel plot and confirmed by the Egger’s linear regression test (Intercept −4.06, t = −0.12, *p*-value = 0.909). The sub-group analysis considering only interventional studies (trials), included 8 datasets, and the pooled ES was −2.63 [(95% CI = −4.06; −1.20), *p*-value < 0.001] based on 2644 participants. High statistical heterogeneity was found (Chi^2^ = 224.80, df = 7, I^2^ = 96.89, *p*-value < 0.001). Potential publication bias was found by the visual assessment of the funnel plot and confirmed by the Egger’s linear regression test (Intercept −5.12, t = −2.56, *p*-value = 0.043).

### 3.5. Subgroup Analysis by Gender

Considering The sub-group analysis considering only women, included seven datasets, and the pooled ES was −1.91 [(95% CI = −2.77; −1.04), *p*-value < 0.001] based on 1415 participants. High statistical heterogeneity was found (Chi^2^ = 217.37, df = 6, I^2^ = 97.24, *p*-value < 0.001). Potential publication bias was found by the visual assessment of the funnel plot and confirmed by the Egger’s linear regression test (Intercept −5.29, t = −3.82, *p*-value = 0.012). The sub-group analysis considering only men, included three datasets, and the pooled ES was −0.11 [(95% CI = −0.38; 0.16), *p*-value = 0.430] based on 928 participants. A high statistical heterogeneity was found (Chi^2^ = 240.33, df = 26, I^2^ = 89.18, *p*-value < 0.001). However, no publication bias was found by the visual assessment of the funnel plot and confirmed by the Egger’s linear regression test (Intercept −1.20, t = −0.99, *p*-value = 0.503).

## 4. Discussion

The current systematic review with meta-analysis—which included 43 studies in qualitative evaluation and 42 studies in the quantitative analysis—provided data on the association between objectively measured PA and the risk of depression. Since some studies expressed data separated for gender, a total of 42 datasets have been considered. The pooled ES based on 37,408 subjects indicated a significantly protective effect of PA on depression [−1.16 (95% CI = −1.41; −0.91), *p*-value < 0.001] while, in the subgroup analysis including only cross-sectional datasets, the risk of prevalent depression was estimated on 17,191 participants and the ES was −0.23 [(95% CI = −0.30; −0.16)]. In subgroup analysis including only longitudinal datasets, the risk of incident depression, estimated on 17,515 participants, was lower −2.61 [(95% CI = −7.41; 2.21).

With the purpose of deeply understanding the strength of the association between objectively measured PA and depression, a sub-group analysis by participants’ comorbidity has been conducted. When studies assessing the association among participants with comorbidities were considered, the ES were not statistically significant (apart for COPD participants). However, prescription of adapted PA among participants affected by co-morbidities should be considered [71]. To the contrary, when only studies with general population (otherwise healthy people) were considered, the pooled ES was statistically significant, indicating an inverse association between PA objectively measured and depression (more PA was associated with lower risk of depression). A subgroup analysis by gender was conducted as well, showing a protective effect of PA only for women. However, this result should be considered carefully, since only three studies assessed PA and depression only in men, reducing the sample size.

These results are extremely important considering that depression is one of the leading causes of disabilities worldwide [1]. In the last fifty years a great concern was casted on physical health of depressed individuals. This could be due because physical exercise seems to improve several biomarkers implicated in depression (e.g., impaired neuroplasticity, autonomic and immune imbalances) [9]. In in-vivo models, physical activity showed a serotoninergic effect as some antidepressant medications [8]. Moreover, PA has demonstrated an effect on inflammatory processes, through the hypothalamic-pituitary-adrenal axis regulation involved in the development of depression [9]. Additionally, higher levels of brain derived neurotrophic factor have been found after physical exercise [10]. Lastly, the level of PA directly affects the upper limit of oxygen uptake which depends on the capacity of the cardiorespiratory system to transport oxygen to the organs, including the brain. A lower oxygenation of the brain may result in a chronic cerebral ischemia and, if the affected areas are involved in a mood regulation, this may increase the risk of depression [12].

In the last decades, several studies have shown that a healthy lifestyle, in particular the intensity and length of physical activity [72,73], are important in the prevention and treatment of depression [7]. In our analysis we could not assess the relation between severity of depression and intensity of PA, as in most of the primary studies included, severity of depression was not reported and PA intensity was expressed using different methods. The results from our review confirm the beneficial effect of PA on depression, especially for participants without comorbidities. In this regard, health education campaigns aimed to promote PA should be fostered [74,75,76], especially because approximately 40% of the adult population worldwide is insufficiently physical active [77]. However, in order to better interpret our results, another important aspect should be considered: indeed, even if several sub-group analyses have been conducted, the value of heterogeneity remained stably high. Although a sensitivity analysis including only datasets with otherwise healthy people has been conducted, the I^2^ remained extremely high. However, a I^2^ value higher than 90% means that heterogeneity is directly due to heterogeneity among studies, instead of sampling error [78]. Moreover, primary papers expressed the level of PA using different types of unit of measures and also the results were reported using different modalities. Even if the pooled ES has been estimated by log OR, allowing comparability, this underlying heterogeneity might have affected the assessment of the I^2^ [79]. Another potential explanation of heterogeneity could be the different type of duration of measurement, the device used and the questionnaire adopted to diagnose depression. Furthermore, a variety of confounding variables were selected in original studies and, in order to control the results, we pooled the models with the highest level of adjustment.

### Limitantions and Strengths

The main limitation of this systematic review is the high I^2^ value that might reduce the generalizability of our results. Most studies are observational and based on cross-sectional analysis. Nevertheless, we performed sensitivity analyses only including trials and longitudinal studies, increasing the robustness of our results. Due to the high heterogeneity in reporting the level of PA performed by participants in original studies, it was not possible to identify a recommended level of PA. The inability to estimate an association between severity of depression and PA is another important limitation. The main strengths of this review are being systematic in nature and its comprehensive way to include the entire scientific evidence published so far on the main medical-scientific databases. Furthermore, the pooled ES was significantly large, based on 37,408 participants, and sub-group analyses have been conducted based on participants’ comorbidity and study design. In the primary studies, diagnosis of depression was consistently based on the DSM criteria and was established by trained investigators using validated assessment scales mainly with interrater reliability.

## 5. Conclusions

To conclude, the results of this systematic review and meta-analysis clearly show a statistically significant protective effect of objectively measured PA on prevalent and incident depression. An increased PA is associated with lower risk of depression. The advantages of our study are several. Firstly, this study offers a systematic overview of previous studies assessing objectively measured PA and depression. Secondly, this study highlights the usefulness of objectively measured PA compared to self-reported one. Objectively measured PA is not only more precise in estimating duration, total amount, and intensity of PA, but indirectly it can also better strengths the association with some diseases, as depression. Thirdly, this study shows the importance to promote physical activity forasmuch it can help to reduce the high burden of depression in our society. Lastly, our findings are relevant for both policy makers and clinicians as physical activity is one of the cheapest, non-pharmacological treatment that might be prescribed to the general population with potentially major public health impact. Physical activity is important across ages and should be integrated into daily life.

## Figures and Tables

**Table 1:** Detailed description of inclusion/exclusion criteria according to a Population, Exposure, Outcomes and Study design (PEOS).

Search Strategy	Details
Inclusion criteria	P: adults (men and women) E: physical activity objectively measured O: Depressive disorder S: Trials, cohort studies, case-control, cross-sectional
Exclusion criteria	P: people < 18 years old E: physical activity not objectively measured (self-reported) O: other psychological disorders S: not original papers (opinion paper, review article, commentary, letter, protocols, article without quantitative data)
Language filter	English
Time filter	No filter (from inception)
Database	PubMed/Medline; EMBASE, Web of Science; Scopus, PsycoInfo, Cochrane

**Table 2:** Descriptive characteristics of the included studies stratified by study design and listed in alphabetical order.

Author Year [Reference]	Country	Characteristics	Study Period	Age and Gender	Sample Size and Gender	Depressed Subjects	Attrition +	Device Used	Duration of Measurement	Tool Used for Depression Diagnosis	PA	Results	QS
**Cross-sectional studies**													
Al-Eisa, 2014 [31]	Saudi Arabia	Female students	2014	Mean: 20.9 ± 1.4 y, F	76	52%	29	Pedometer	3 weeks	BDI-II	PA = 8715 steps/day	R = −0.78 *p* ≤ 0.01;	4
Alosco, 2012 [32]	USA	Persons with heart failure	*n*.a.	Mean: 68.81 ± 8.8 y, M, F	96 (M = 63, 5%, F = 36, 5%)	*n*.a.	27	GT1M+ accelerometer (ActiGraph, Pensacola, Florida)	7 days at baseline, 3 months, 12 months	BDI-II	MVPA = 3.24 ± 9.0 min/day	β = −64.35 *p* < 0.05 t = −2.32	6
Altenburg, 2013 [33]	The Netherlands	Patients with stable chronic obstructive pulmonary disease (COPD)	*n*.a.	Mean: 62 (54–69) y M, F	155 (M = 102, F = 53)	*n*.a.	0	Yamax-Digiwalker pedometer (SW-200)	2 weeks	HADS	PA = 4206 (2387–6284) steps/day	R = −0.191 *p* < 0.05	4
Arrieta, 2018 [34]	Spain	Partecipants from nursing home	October 2016–June 2017	84.9 ± 6.9 years	114 (81 F, 33 M)	25% (at risk of depression	0	Actigraph GT3X model	7 days	GDS	MVPA = 0.9 ± 1.2 min/day	β = 1.142 *p* = 0.028	7
Bade, 2018 [35]	USA	Lung Cancer Patients	2014–2015	Mean: 66 ± 7.75(SD) y (51–80) M, F	30 (M = 20, F = 10)	*n*.a.	43	Accelerometer (Fitbit Zip)	7 days	PHQ-9	PA = 4877 ± 305	R = −0.40	5
Barriga, 2014 [36]	Portugal	COPD patients	*n*.a.	Mean: 67 ± 9.6 y, M	55 (sex *n*.s.)	*n*.a.	0	Pedometer	Number of steps per day, on three consecutive days	HADS	PA = 4972.4 ± 2242.3	R = −0.424 *p* < 0.01	3
Di Marco, 2014 [37]	Italy	COPD patients	*n*.a.	Mean: 71 ± 6 y M, F	70 (M = 52, F = 18); No Depression = 51 (18% F)	19 (47% F)	0	Accelerometer (SenseWear Pro Armband, BodyMedia)	5 days	HADS	No Depression PA = 6950 ± 2431 Depresed PA = 5055 ± 2576	β = 0.106 *p* = 0.84	6
Dillon, 2017 [38]	Ireland	Patients in the 50–69 year age group.	2011	Mean: 59.6 ± 5.5 y M, F	397 (M = 182, F = 214)	18.2%	78	Accelerometer (ActivInsights Ltd.)	7 days	CESD-20	Mean Light PA No Depression = 103 min/day Depressed = 105 min/day	β = −0.34 (95% CI = −0.64 to −0.04)	7
Drieling, 2014 [39]	USA	Obese latino immigrants	July 2009–September 2010	*n*.a.	207 (48 M, 159 F)	36.7%	0	Pedometer	7 days	CESD	6.3 ± 3.1 steps/day in thousands	β = −0.02 SE 0.01 *p* = 0.03	6
Elbelt, 2015 [41]	Germany	High grade obesity	2008–2010	Mean: 42 ± 12 y	50 (10 M, 40 F)	36%	0	Accelerometer	3 days	PHQ-9	No depressed: 6023 ± 2459 steps/day Depressed: 6532 ± 3085 steps/day	r = 0.023	7
Fenton, 2017 [42]	England	Rheumatoid Arthritis patients	*n*.a.	Mean: 54.92 ± 12.39 y	61 (F = 67.2%)	*n*.a.	36	Actigraph GT3X+, accelerometer (Pensacola, FL)	7 days	HADS	LPA = 269.35 ± 69.35 min/day	β = −0.30 *p* < 0.05	10
Gaskin, 2016 [44]	Australia	Prostate cancer survivors	*n*.a.	65.6 ± 8.5 y	98 (M)	*n*.a.	*n*.a.	ActiGraph GT1 M (Pensacola, FL)	7 days	CESD	MVPA = 38 min/day	β = ·0.00 *p* = 0.97	10
Howie, 2018 [49]	Australia	Subsample of the 22th follow-up measurement of the Raine cohort Study.	2011	*n*.a.	475 (256 F, 219 M)	1.3%	299	Actigraph GT3X+, accelerometer Pensacola, FL	7 days	DASS-21	MVPA F = 27 min/day M = 34.1 min/day	F: RR = 0.99 (95% CI = 0.98–1.00), *p* = 0.078 M: RR= 1.01 (95% CI = 0.99–1.02), *p* = 0.300	10
Huong, 2013 [58]	USA	COPD patients	*n*.a.	Mean: 66.5 ± 8.8 y M, F	148 (M = 115, F = 33)	29%	0	Accelerometer Stepwatch 3 Activity Monitor (OrthoCare Innovations LLC)	7 days	HADS	Mean = 6.079 ± 3718	β = −0.19 *p* = 0.02)	7
Jung, 2018 [50]	Japan	Community-dwelling older Japanese adults.	2013	Mean: >75 y M, F	3054 (M = 1491, F = 1563)	598	2.203	Accelerometer (GT40-020)	7–40 days	GDS	No Depression = 5059.6 ± 53.7 steps/day Depressed = 5003.0 ± 112.1 steps/day	Coehns = 0.03 *p* = 0.359	7
Kangasniemi, 2014 [51]	Finland	Adults, general population	2011	Mean 43 ± 5.2 y,	108 (58 F, 50 M)	*n*.a.	109	ActiGraph-GT1M, accelerometer LLC, Pensacola, Florida	7 days	BDI- II	Less Active: 24.3 ± 12.4 min/day More active: 62.7 ± 24.7 min/day	r = −0.24, (95% CI 0.38, 0.08)	7
King, 2014 [52]	USA	Adults with ≥class 2 obesity.	2009	Mean 45 (18–78) y	850 (673 F, 177 M)	31.8%	3626	StepWatch™ 3 Activity Monitor (OrthoCare Innovations, Washington, D.C.)	7 days	BDI- II	PA ≥ 1000 steps/day Mean: 7321.0 steps/day	OR = 1.03 (95% CI 0.97–1.09)c	7
Loprinzi, 2012 [54]	USA	non-institutionalized U.S. civilians	2005–2006	48.4 ± 0.8 y	1146 (611 M)	9.5%	*n*.a.	ActiGraph AM-7164, accelerometer Walton, Beach, FL.	7 days	PHQ-9	MVPA = 2020–5998 steps/min	M: OR 0.71 (95% CI 0.53–0.95) F:OR = 0.74 (95% CI 0.57–0.96)	10
Loprinzi 2013 (A) [55]	USA	non-institutionalized USA civilians	2006	Mean: 73.5 ± 0.2 y	708 (57.2% M)	14.9%	*n*.a.	ActiGraph AM-7164, accelerometer Walton, Beach, FL.	7 days	PHQ-9	MVPA = 10.0 ± 0.9 min/day	OR = 0.78 (95% CI 0.64–0.94)	9
Loprinzi, 2013 (B) [56]	USA	Diabetic non-institutionalized USA civilians	2006	Mean: 59.6 ± 1.2 y	372 (51.4% F)	3.1%	*n*.a.	ActiGraph AM-7164, accelerometer Walton, Beach, FL.	7 days	PHQ-9	MVPA = 12.2 ± 1.3 min/day	β = −0.03 (95% CI −0.05—−0.006) *p* < 0.05	10
Ludwig, 2018 [57]	UK	UK residents	2013–2015	69 ± 4.1 y	1720 (M = 85.5%)	4%	20	ActiGraph GT3X accelerometer (ActiGraph, Florida, USA)	7 days	PHQ-9	PA = 6151 steps/day	β = −0.170 *p* < 0.001	7
Park, 2017 [60]	UK	Subjects living facilities across England	*n*.a.	77.5 ± 8.2 y	85 (M = 31.8%)	*n*.a.	0	GT3X+, WGT3X-BT; ActiGraph, Pensacola, FL, USA	*n*.a.	HADS	MVPA = 9.74 min/day	Χ^2^ = 8.45 *p* = 0.004	5
Song, 2011 [64]	USA	community residents older than 20 years	2006	≥20 y	4058 (51.32% F)	19.5%	6290	ActiGraph^®^ AM-7164, accelerometer Walton, Beach, FL.	7 days	PHQ-9	MPA = 30 min daily and more than 3 days a week	OR = 0.72 (95% CI 0.54–0.97) *p* < 0.05	7
Vallance JK, 2011 [66]	USA	non-institutionalized civilian US citizens	2005–2006	45.7 ± 13.7 y	2862 (1417 M)	195	*n*.a.	ActiGraph AM-7164, accelerometer Walton, Beach, FL.	7 days	PHQ-9	MVPA = 20.2 ± 0.2 min/day	OR = 0.37, (95% CI, 0.20 to 0.70) *p* < 0.01	9
Vallance J.K, 2015 [67]	Canada	Colon cancer survivors	*n*.a.	Mean: 64.3 ± 10.3 y M, F	180 (M = 99, F = 81)	8.5%	17	Actigraph GT3X+ accelerometer	7 days	PHQ-9	non-extrapolatable	non-extrapolatable	8
Whitaker, 2014 [69]	USA	Overweight and obese women	*n*.a.	Mean: 38.3 ± 7.6 y	196 (F)	*n*.a.	34	ActiGraph-GT1M, accelerometer LLC, Pensacola, Florida	7 days	CESD-10	MVPA ≥ 2400 steps/min	t = 0.30 *p* = 0.77	9
**Case-control studies**													
O’Brien JT, 2016 [59]	UK	adults > 60yo	2015	74 ± 6 y	58 (43 F)	29	0	Accelerometer	7 days	Montgomery–Åsberg Depression Rating Scale (MADRS); GDS-15	0.17 acceleration/min/day	r = −0.37 *p* ≤ 0.05	7
**Cohort studies**													
Duenas-Espin, 2016 [40]	Europe (Athens, Leuven, London, Groningen).	COPD patients	July–November 2011	M, F Mean: 67 ± 8y	220 (149 M, 71 F)	5%	*n*.a.	Accelerometer Dynaport MoveMonitor (McRoberts BV, The Hague, the Netherlands).	7 days at baseline, 6 and 12 months	HADS hospital anxiety and depression scale) (depression>11 points)	4812 ± 3147 steps/day	β = 0.6 (95% CI 0.5 to 0.8) *p* = 0.01	5
							**Follow-up** = 1 y						
Po-Wen, 2017 [53]	Taiwan	community-dwelling older adults	2012–2014	Mean: 74.5 y M, F	285 (M = 125, F = 149)	*n*.a.	11	ActiGraph GT3X-BT (ActiGraph, Pensacola, FL)	7 day at baseline	15-item Geriatric Depression Scale	MVPA>1951 steps/min	RR: 0.88 95% CI (0.79–0.98) *p* = 0.021	8
							**Follow-up** = 2 y						
Raudsepp, 2017 [61]	Estonia	generally healthy community-dwelling individuals aged 67–74 years	2011–2013	67–74 y M, F	195 (M = 85, F = 110)	*n*.a.	23	Yamax-Digiwalker pedometer (SW-200-024)	1 week each year, per 3 years	15-Item Geriatric Depression Scale	6394.5 daily walking steps	β = −0.17 Χ2 = 83.27	6
							**Follow-up** = 3 y						
Rethorst, 2017 [62]	USA	Hispanic/Latino men and women, age 18 to 74 years at time	2008–2011	Mean: 41.06 ± 0.25 y M, F	16,415 (52.13% F)	*n*.a.	*n*.a.	Actical B-1 version accelerometer	7 days at baseline	Center for Epidemiological Studies Depression Scale 10	VPA≥3962 steps/min	β = −0.936	4
							**Follow-up** = 7 days						
Sylvester, 2017 [64]	Canada	Breast cancer women over 1 year post-treatment	*n*.a.	55.01 ± 10.96 y	201 F	*n*.a.	0	ActiGraph GT3X-BT (ActiGraph, Pensacola, FL)	7 days every 3 months	10-item Center for Epidemiologic Studies Depression Scale	MPA = 14.73 ± 11.6 min/day	β = −0.73; *p* = 0.03	8
							**Follow-up** = 1 y						
Trinh, 2015 [65]	Canada	Patients with breast cancer in stage I–III without metastatic disease	2010–2012	Mean: 55 ± 11 y F	199 (F)	*n*.a.	4	ActiGraph GT3X-BT (ActiGraph, Pensacola, FL)	7 days at baseline	CES-D10	MVPA mean 107.1 ± 81.3 min/week)	β = −0.10 *p* = 0.19	4
							**Follow-up** = 7 days						
**Trial studies**													
**Author** **Year**	**Country**	**Characteristics**	**Study Period**	**Age and Gender**	**Sample Size**	**Depressed Subjects**	**Attrition +**	**Device Used**	**Duration of Measurement**	**Tool Used for Depression Diagnosis**	**PA**	**Results**	**Follow-up**
Abedi, 2015 [30]	Iran	Post-menopausal women	*n*.a.	*n*.a.	106 F	*n*.a.	*n*.a.	Pedometer	12 weeks	BDI-II	Before 76,377 steps/months; after: 106398/month	Intervention vs. control group 13.7 ± 5 vs. 19.6 ± 4.79 *p* < 0.001	12 weeks
Freitas, 2018 [43]	Brazil	Obese adults with asthma	*n*.a.	30–60 y	51 F	58.8%	*n*.a	ActiGraph GT3X-BT (ActiGraph, Pensacola, FL)	7 days	HADS	Training group (after): 10,000 steps/day Control group(after): ~8000 steps/day	r = 0.52 *p* < 0.01	3 months
Golsteijn, 2018 [45]	Holland	prostate and colorectal cancer patients survivors	2015–2016	66.5 ± 7.1 y	427 (M, F)	*n*.a.	na	ActiGraph GT3X-BT (ActiGraph, Pensacola, FL)	7 days	HADS	MVPA > 3 MET MVPA = 271 ± 211 min/week	β = −0.64 *p* = 0.005	6 months
Hallam, 2018 [46]	India, Australia, and 21 other countries	General Population, of Stepathlon corporate challenge	2015/16	16–74 y	1963 (1458 M, 505 F)	*n*.a.	na	own personal pedometer, or activity monitoring device	100 days	DASS	*n*.a.	r = − 0.026 *p* = 0.254	100 days
Hartescu I, 2015 [47]	UK	Inactive people with insomnia	2014	59.8 ± 9.46 yo	41 (30 F, 11 M)	*n*.a.	*n*.a.	ActiGraph GT3X-BT (ActiGraph, Pensacola, FL)	6 months	BDI-II	Intervention group 66.50 ± 30.37 (min per week)	Cohen: 0.87 (0.19–1.56)	6 months
Hospes G, 2009 [48]	Netherlands	COPD patients	2008	63.1 ± 8.3 y	35 (21 M)	*n*.a.	*n*.a.	Pedometer Digiwalker SW-2000 (Yamax; Tokyo, Japan)	12 weeks	BDI-II	Intervention group Before 7087 ± 4058 After 7872 ± 3962	β = 0.93 *p* = 0.01	12 weeks
van den Berg-Emons, 2004 [68]	Netherlands	Patients with stable chronic heart failure	*n*.a.	58.6 ± 12.1	34 (25 M e 9 F)	*n*.a.	*n*.a.	Accelerometer (AM, Temec Instruments, Kerkrade	48 h	HADS	Intervention group: 9.9% (of 24 h) Control group: 7.4%	Intervention group: 3.4(±4.0); Control group: 4.8 ± (3.1)	3 months
Vetrovsky T, 2017 [70]	Czech Republic	inactive people from general population in primary care setting	2015	41 ± 10 y	23 (12 M, 11 F)	0 at baseline	0	tri- axial pedometer (eVito 3D Step Counter SL; HMM Diagnostics GmbH, Dossenheim, Germany)	7 days	HADS	After = +1676	Mean difference = −2.4 [95% CI −3.7, −1.2] *p* = 0.001	3 months

+ Number of subjects lost or incomplete data; *n*.a. not available; *n*.s. not specified; QS = quality score; COPD Chronic obstructive pulmonary disease; UK United Kingdom; USA United States of America; MVPA moderate-to-vigorous physical activity; M male; F female; BDI-II Beck Depression Inventory-II; HADS Hospital Anxiety and Depression Scale; GDS Goldberg Depression Scale; Center for Epidemiologic Studies for Depression Scale CESD-10; PHQ-9 Patient Health Questionnaire-9; DASS-21 Depression Anxiety Stress Scales.

## Supplementary Materials

The following are available online at https://www.mdpi.com/1660-4601/17/10/3738/s1, F, Table S1: Search strategy in PubMed/MEDLINE, Table S2: Assessment of risk of bias for trials, using The Cochrane Collaboration’s.
