# Supplementary material for: Depression and Objectively Measured Physical Activity: A Systematic Review and Meta-Analysis
Source: Int J Environ Res Public Health. 2020 May 25;17(10):3738. doi: 10.3390/ijerph17103738 (PMC7277615; doi:10.3390/ijerph17103738)
Supplement: Supplementary file 1 [file ijerph-17-03738-s001.pdf]

Review

Depression and Objectively Measured Physical Activity: A Systematic Review and Meta-Analysis

Vincenza Gianfredi, Lorenzo Blandi, Stefano Cacitti, Mirko Minelli, Carlo Signorelli, Andrea Amerio and Anna Odone

Supplementary Table S1. Search strategy in PubMed/MEDLINE.

SET	PubMed
1	"Patient Health Questionnaire"[Mesh]
2	"Depressive Disorder, Major"[Mesh]
3	"Dysthymic Disorder"[Mesh]
4	"Depressive Disorder"[Mesh]
5	"Depression"[Mesh]
6	depression[Title/Abstract]
7	depressive[Title/Abstract]
8	PHQ[Title/Abstract]
9	Sets 1-8 were combined with "OR"
10	"Screen Time"[Mesh]
11	"Exercise"[Mesh]
12	"Sports"[Mesh]
13	"Leisure Activities"[Mesh]
14	"Sitting Position"[Mesh]
15	"Supine Position"[Mesh]
16	"Sedentary Behavior"[Mesh]
17	("Walking"[Mesh]
18	"Walking Speed"[Mesh]
19	"Bicycling"[Mesh]
20	"Endurance Training"[Mesh]
21	"Movement"[Mesh]
22	Movement*[Title/Abstract]
23	sport*[Title/Abstract]
24	physical activity[Title/Abstract]
25	physical activities[Title/Abstract]
26	physical exercise[Title/Abstract]
27	physical exercises[Title/Abstract]
28	walking[Title/Abstract]
29	stepping[Title/Abstract]
30	sitting[Title/Abstract]
31	Sets 9-30 were combined with "OR"
32	"Ergometry"[Mesh]
33	"Physical Endurance"[Mesh]
34	"Accelerometry"[Mesh]
35	pedometer*[Title/Abstract]
36	device*[Title/Abstract]
37	"quantitative measurement"[Title/Abstract]
38	"quantitative measurements"[Title/Abstract]
39	"objective measurement"[Title/Abstract]

40	"objective measurements"[Title/Abstract]
41	"objectively measured"[Title/Abstract]
42	Set 32-41 were combined with "OR"
43	"Review" [Publication Type]
44	"Review Literature as Topic"[Mesh]
45	review[Title/Abstract]
46	"Meta-Analysis" [Publication Type]
47	"Meta-Analysis as Topic"[Mesh]
48	"Network Meta-Analysis"[Mesh]
49	"Infant, Newborn"[Mesh]
50	"Infant"[Mesh]
51	"Child"[Mesh]
52	child[Title/Abstract]
53	children[Title/Abstract]
54	infant*[Title/Abstract]))
55	"Depression, Postpartum"[Mesh]
56	Sets 43-55 were combined with "OR"
57	Sets 9, 31 and 42 were combined with "AND"
58	Sets 57 and 56 were combined with "NOT"
59	Set 58 was limited to English language
	Grey lines show how the listed keywords were combined

Supplementary Table S2. Assessment of risk of bias for trials, using The Cochrane Collaboration's tool.

Author year	Selection	Performance *	Detection *	Attrition	Reporting
Abedi et al. 2015	Low	High	High	Low	Low
Freitas et al. 2018	Low	High	High	Low	Low
Golsteijn et al. 2018	Low	High	High	Low	Low
Hallam et al. 2018	High	High	High	High	Low
Hartescu et al. 2015	Low	High	High	Low	Low
Hospes et al. 2009	Low	High	High	Low	Low
Van der Berg-Emons et al. 2004	Low	High	High	Low	Low
Vetrovsky et al. 2017	High	High	High	Low	Low

In physical activity interventional studies neither participants and researchers could be blinded, due to study design.
